# Paediatric mass casualty response through the lens of Functional Resonance Analytical Methodology- lessons learned

**DOI:** 10.1186/s13049-024-01264-4

**Published:** 2024-09-20

**Authors:** R. J. MacKinnon, D. Slater, R. Jenner, T. Stenfors, C. Kennedy, K. P. Härenstam

**Affiliations:** 1 Department of Paediatric Anaesthesia, Royal Manchester Children’s Hospital, Manchester, UK; 2 School of Engineering, Cardiff University, Cardiff, UK; 3 Department of Paediatric Emergency Medicine, Royal Manchester Children’s Hospital, Manchester, UK; 4 Department of Learning, Informatics, Management and Ethics, Karolinska Institutet, Stockholm, Sweden; 5 Paediatric Emergency Department, Children’s Mercy Hospital Kansas City, Kansas City, USA; 6 Paediatric Emergency Department, Karolinska University Hospital, Solna, Sweden

**Keywords:** Mass casualty incident, Paediatric, Patient safety, Resilience engineering, Trauma care

## Abstract

**Background:**

Mass Casualty Incidents are rare but can significantly stress healthcare systems. Functional Resonance Analytical Methodology (FRAM) is a systematic approach to model and explore how complex systems adapt to variations and to understand resilient properties in the face of perturbations. The aim of this study was to use FRAM to create a model of a paediatric trauma system during the initial response to the Manchester Arena Attack to provide resilience-based insights for the management of future Mass Casualty Incidents (MCI).

**Methods:**

Qualitative interviews in the immediate aftermath of a terrorist bombing, were followed up with further in-depth probing of subject matter experts to create a validated and verified FRAM model. This model was compared with real incident data, then simplified for future studies.

**Results:**

A Work As Imagined (WAI) model of how a paediatric emergency department provided resilient healthcare for MCI patients from reception and resuscitation to definitive care is presented. A focused model exploring the pathway for the most severely injured patients that will facilitate the simulation of a myriad of potential emergency preparedness resilience response scenarios is also presented.

**Conclusions:**

The systematic approach undertaken in this study has produced a model of a paediatric trauma system during the initial response to the Manchester Arena Attack, providing key insights on how a resilient performance was sustained. This modelling may provide an important step forward in the preparedness and planning for future MCIs.

## Introduction

On the evening of the 22nd of May 2017, a terrorist denoted an improvised explosive device in the foyer of the Manchester Arena as concert goers, children and adults emerged, killing 23 people (including the attacker). Mass Casualty Incidents (MCI) are defined as incidents which generate more patients at one time than locally available resources can manage using routine procedures. They require exceptional emergency arrangements and additional or extraordinary assistance [1]. MCIs are rare in the context of an individual clinician or institution, but children are often involved when MCI occur [2]. A paediatric MCI should provide an opportunity to explore optimal human and organisational performance, to apply that learning to improve future patient outcomes. Resilience defined as “the intrinsic ability of a system to adjust its functioning prior to, during, or following changes and disturbances so that it can sustain required operations, even after a major mishap or in the presence of continuous stress” [3], is an essential prerequisite of a Major Trauma Centre (MTC). A MTC is a complex socio-technical healthcare system designed to respond effectively to a myriad of clinical scenarios, within which healthcare staff work adaptively to provide patient care. In the immediate aftermath of the Manchester Arena Attack the nearby paediatric MTC demonstrated both resilient elements and a series of successful adaptations to improve patient outcomes during the MCI [4].

During the initial response to the attack twenty-two children aged between eight to fifteen years and five parents presented with blast injuries to the paediatric MTC [5]. One child died in the Paediatric Emergency Department (PED), fourteen children were admitted, four going directly to the operating theatres and six to the Paediatric Intensive Care Unit (PICU).

MCI involving children are rare events [6]. However, learning from such experiences, is a fundamental element of resilience [7]. A lack of in-depth learning after events, severely hampers the capability to respond to future MCIs that may present to a UK MTC. MCI are rare occasions which creates challenges for both individual, team and organisational learning. Modelling is one approach to support learning, with a model being a formal system that can be used to express or represent the “objects and their relationships in the world” that are being investigated [8].

**Fig. 1:**
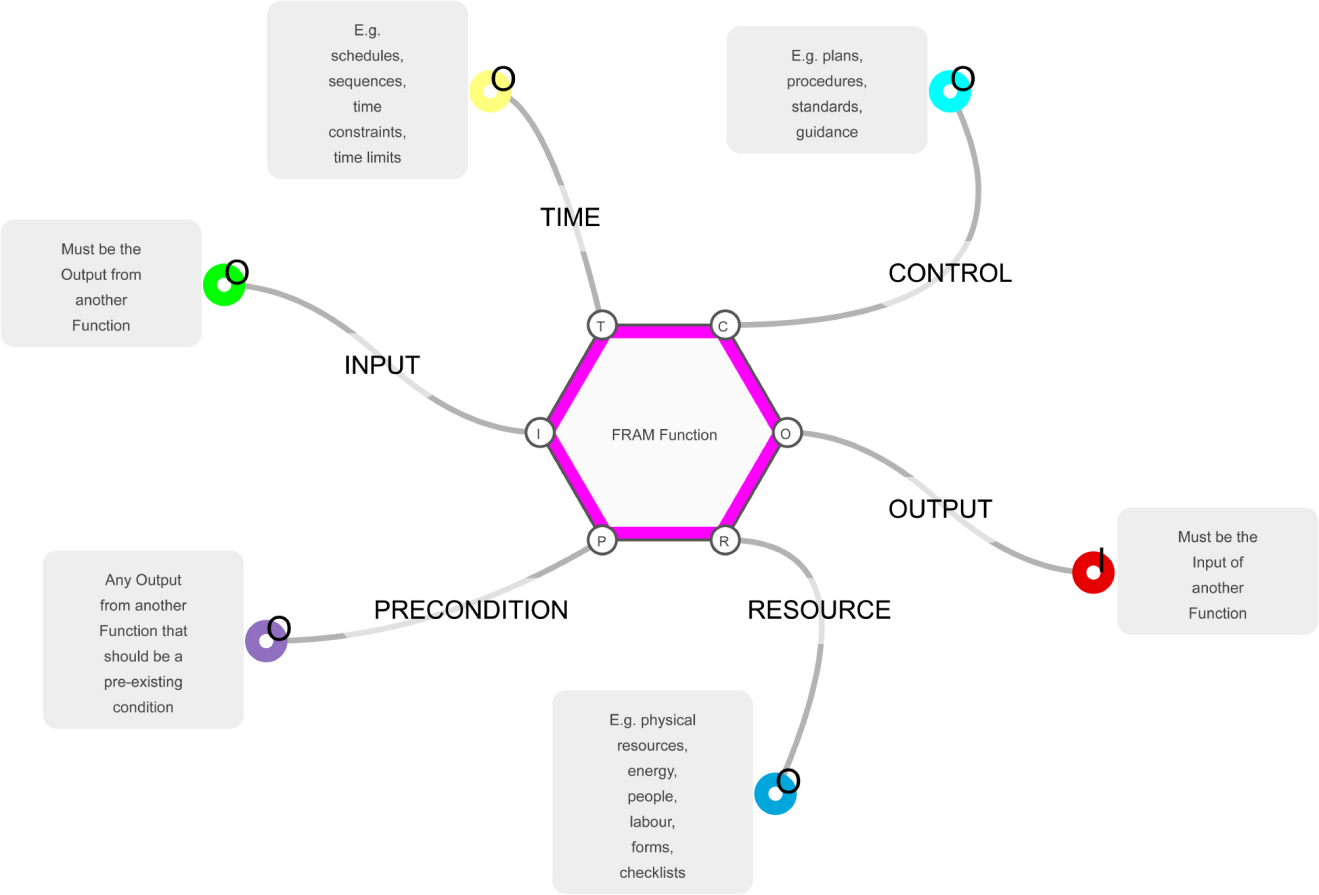
The FRAM Function. (adapted from Hill & Slater, 2023) [10]

Functional Resonance Analytical Methodology (FRAM) facilitates the modelling of complex adaptive systems [9]. A FRAM model is composed of interconnected functions, to do tasks, that describes a complex adaptive system where humans interact with technology. Each function is described in terms of its “aspects” of input, outputs, preconditions for the task to occur, resources used up during the task, conditions that control the function and time considerations for the function to occur (Fig. 1) [9]. FRAM models depict complex systems as composed of a cloud of interlinked functions (tasks) represented as hexagons. The model commences with a function which defines the start of the model, the output of this function fires the input of the next function, the aspects of precondition, resources, time and control can all influence the passage across each function from input to output. The system is then depicted electronically as a cascade of functions from inputs to outputs to the final functions of a model.

FRAM models are used for risk management, accident investigation as well as visualising system interdependencies for prospective analyses in aviation, off-shore oil and maritime industries as well as in healthcare [11]. A FRAM model of the paediatric MTC response to Manchester Arena Attack could provide valuable learning insights into how resilient behaviour was achieved. Such a model would also constitute the foundation stone for predicting how the MTC could respond to differing mass casualty scenarios. Thus, the aim of this study was to use FRAM to create a model of a paediatric trauma system during the initial response to the Manchester Arena Attack to provide insights on resilient management of future mass casualty incidents.

## Methods

### Ethics approval

Local institutional and UK Health Research Authority approval was provided. This study was exempt from ethics review as no patients were involved. As this study involved staff, informed consent was sought and was provided by all participants before participation in all interviews.

### Data collection

Data was collected from two sources: i). **Face to face interviews** of staff members (approximately 20% of the staff working on the night of the attack in PED, Radiology, Critical care and Theatres). Individuals interviewed were asked a standardised open question to ask them to describe how they overcame the challenges that the MCI presented to them. The staff interviewed had diverse roles, within the confines of the paediatric hospital during the attack, ranging from Consultant Surgeon to Hospital Chaplain to Laboratory staff. Interviews were conducted within seven days of the event, as previously described [4]. After the first stage interviews key functions of the hospital e.g. “To triage patients” became apparent. These key functions were informative for the next stage of in-depth interviews conducted with six members of staff, also all working in the hospital on the night of the event, particularly with P1 patients. At these interviews, the interviewees were invited to describe the hospital system and the processes of care of patients from notification of the incident to definitive care (operating theatre, intensive, high-dependency or ward care). When discussing any of the key functions that the initial interviewees or themselves highlighted these six interviewees were directly prompted to discuss any controlling factors, preconditions, timings or resources utilised during these processes of care or tasks. By this approach all of the aspects, that is inputs, outputs, preconditions that had to be there, resources used up, controlling factors and time considerations could be collected for each key stage function in the future model. Each interview lasted approximately one hour. All interviews were audio-recorded and transcribed verbatim. The same trained interviewer (RM) conducted all interviews.

ii) **Document analysis** of the hospital Major Incident Plan documents and iii)**Key process timings at the time of the Arena Attack**, At the time of the Arena Attack the hospital did not have an electronic patient record, the reliable Work As Done (WAD) data was taken from actual timings of patient arrival in to the Paediatric Emergency Department on the patient notes, time stamps on CT scans and times of the patients entering and leaving theatre from the theatre database software.

### Analysis

The initial interviews transcripts were analysed independently by two researchers (RM & DS) to gain a thorough overview of the system. The same two researchers analysed the key timings using a Microsoft Excel spreadsheet and reviewed all the MIP documentation to determine functions that were evident on the night of the attack and not described in the MIP.

Key stages during the flow of patients in the MTC system were identified from all of the first set of interviews and included notification, hospital preparation, patient reception and resuscitation to radiological scanning then definitive care. Each of these stages were probed at the six in-depth follow-up interviews to understand the key functions and the respective aspects, at each stage. The same two researchers independently read these interview transcripts and notes, then met and constructed the initial FRAM model. This model was then iteratively re-presented to the in-depth interview interviewees to verify the model.

### Validation and verification of the FRAM model

Construct validity was assured by the sound system theory basis of FRAM and over a decade of highly credible research [11, 12]and by comprehensively following established FRAM approaches [9] with experienced FRAM analysts DS & RM. Content validity was maintained by the face validity of using subjective evaluation through the above iterative interviews [4], and discussions within the research group as well as with external colleagues who have a deep knowledge of the events under study [13].

Verification of the FRAM model was achieved utilising the FRAM Model Interpreter which formally checks the model consistency and correctness in a logical stepwise process to ensure completeness, and no dead ends or orphan functions [14].

Further verification of the model was achieved by exploring some actual timings during the MCI and comparing these with those produced by the model using expected timings for functions, as detailed below.

### A focussed model of work as imagined (WAI) for future analyses

Two members of the research team (RM & DS) agreed upon the key functions of interest. The remaining functions were removed from the model and the model integrity was verified as above with the FRAM Model Interpreter [14].

## Results

### A FRAM model of a paediatric MCI

**Fig. 2:**
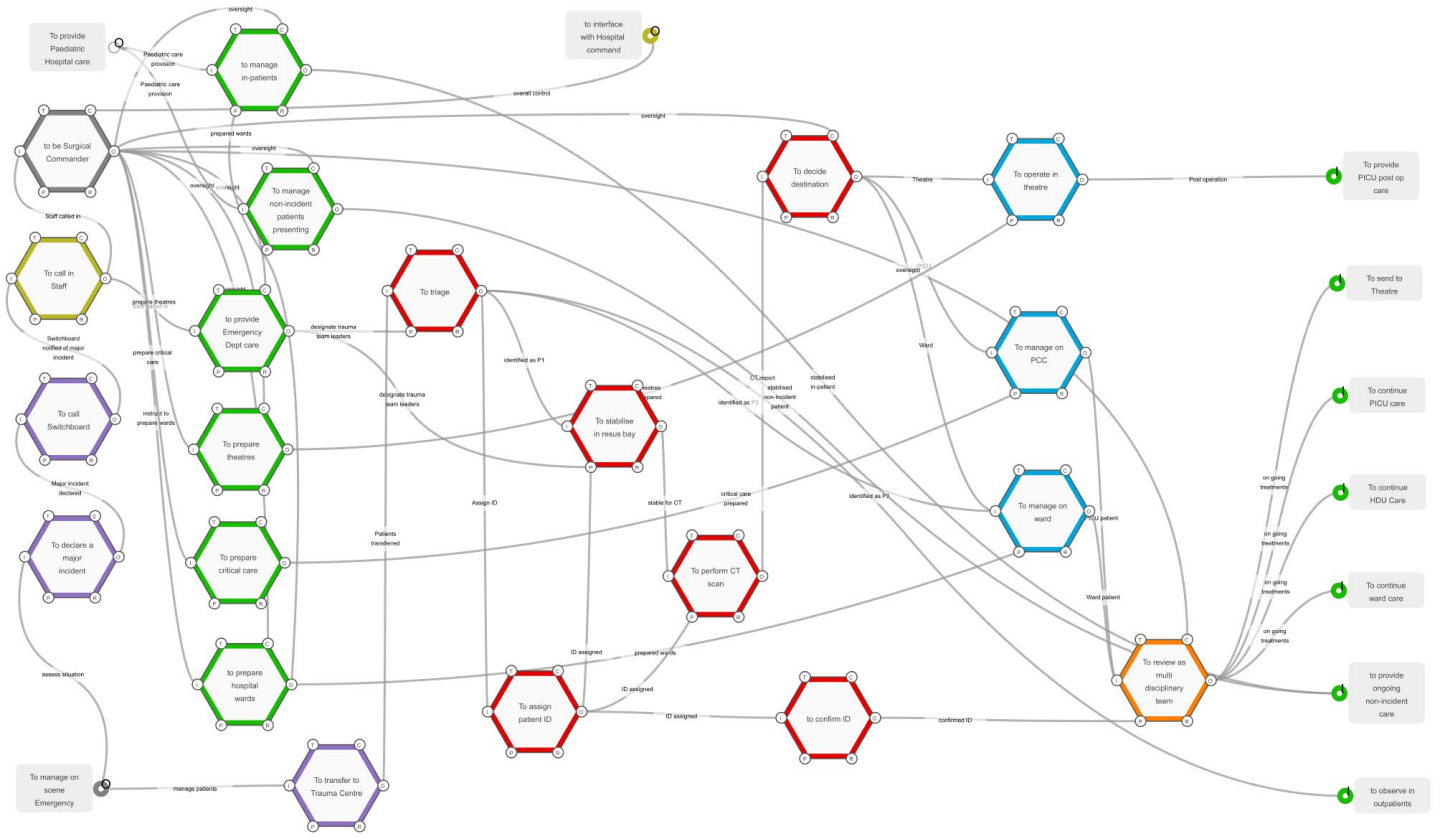
A FRAM model of the essential functions of a paediatric major trauma centre responding to a mass casualty event

The primary result of this study is the creation of a verified and validated FRAM model of how the paediatric major trauma system functioned during the mass casualty event as shown in Fig. 2.

The functions and how they are “coupled” (inter-connected) are represented by the cloud of hexagons and interconnected lines. The model commences with a function outside of the system studied “To manage the on-scene Emergency”, (at the bottom left of the model). This function is termed a background function, shown as a grey box, this function is outside the boundary of the system of interest and activates the model to begin. Other background functions, also coloured grey include “To Provide Paediatric Hospital Care”, referring to ensure care is given to all the other none-mass casualty incident children and “To interface with Hospital Command”, the linkage to the hospital command and control structure during the event. The boundary of study of the model is then completed by a series of final functions, also coloured grey, “To provide PICU post op care”, “To send to Theatre”, “To continue PICU care”, “To continue HDU (High Dependency Unit) care”, “To continue ward care” “To provide ongoing non-incident care” and “to observe in outpatients”.

Each of the stages of the care pathway is given a different colour, notification functions are purple, staff call in is yellow, creation of a surgical commander silver, hospital preparation functions are green, the reception, resuscitation, and radiology functions are red, to manage in theatre or Paediatric Critical Care (PCC) or the wards are blue and the multi-disciplinary ward round is orange.

Table 1 maps the key functions against the resilience potentials of to respond, monitor, anticipate and learn [6]. Most of the key functions of the complex system are responsive in nature, with staff being called in, then a one-way flow of stabilising patients in resuscitation bays, transferring the patients to the Computer Tomography (CT) scanner, then deciding where the patient should go to next, either directly to theatre, to the PICU or HDU or the wards.

Monitoring functions within the system included “To be the Surgical Commander” and “To review (patients) as part of a multi-disciplinary ward round”. The “To be Surgical Commander” was also one of two anticipatory functions alongside “To decide” the (patient) destination. The only learning resilience function identified in the system was the “To review as part of a multi-disciplinary team”.

**Table 1:** Table of key functions of the FRAM model and resilience potentials

Key functions	Respond	Monitor	Anticipate	Learn
To call staff in	X			
To be surgical commander		X	X	
To stabilise in resus bay	X			
To perform CT scan	X			
To decide destination	X		X	
To review as multi-disciplinary team		X		X

### Validation of the FRAM model

With confidence developed in the model, actual timings during the MCI were compared with those produced by the model using expected timings for functions. These expected WAI findings were Function Process Time (Tp) the time it took for a function to go from input to output, the WAI Function Output Lag Time (To) the time it took to move from one function ending to starting another function and WAI Total Time of Functions (Tt) the total time for functions in the system. These expected timings were constructed on discussion with subject matter experts, for example discussion with senior PED nurse regarding how many minutes it takes to triage a severely injured child. The exception was the function “To stabilise in Resus” which was theoretically derived from a series of simulated resuscitations suggesting an average resuscitation time of thirty minutes for trauma patients published previously [15]. Work As Done (WAD) in FRAM models represents the actual work done within the system of interest, as opposed to how it is imagined to work (WAI). Mean WAD Function Start Times and Function Process Times are presented. Table 2 shows the expected mean timings produced by the model of the MCI and timings recorded during the MI for the first eight patients, three of whom went to theatre.

**Table 2:** Expected mean process timings from FRAM model and mean actual process timings of MI

FRAM model Function	WAI Function Process Time Tp (min)	WAI Function Output Lag To (min)	WAI Total Time of Functions Tt (min)	Mean WAD Function Start Time from patient arrival (min)	WAD Function Process Time (min) *n* = 3
To triage (*n* = 8)	1	1	2		
To stabilise in Resus (*n* = 8)	30	2	32		
To perform CT scan (*n* = 8)	15	1	48	37	
To decide destination (*n* = 8)	5	5	58		
To operate in Theatre (*n* = 3)	60	10	141		139
To commence post op PICU care (*n* = 3)					307

**Fig. 3:**
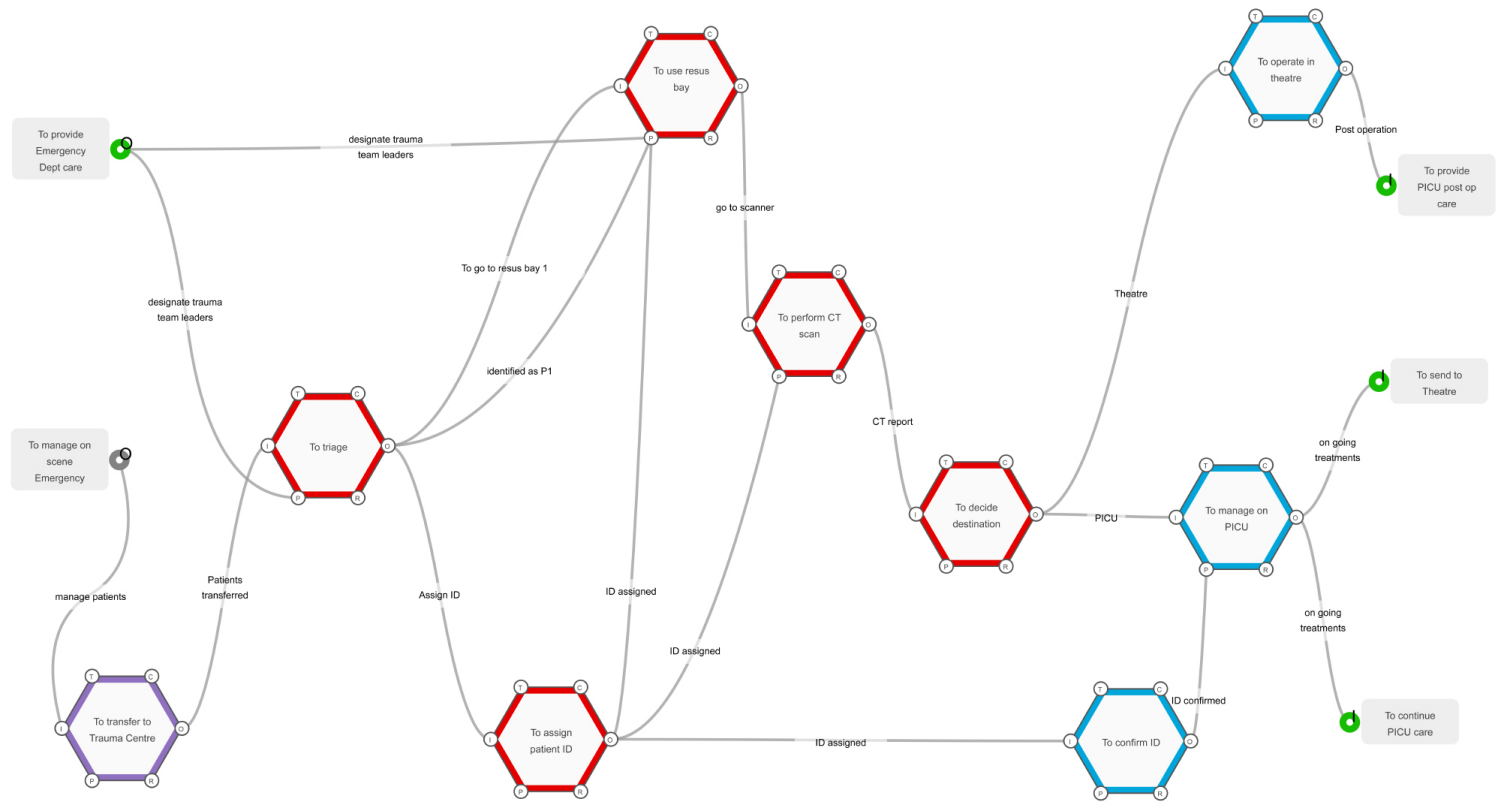
A simplified FRAM model of a paediatric major trauma centre responding to a mass casualty event

The final stage of the modelling was to focus the WAI Trauma system studied (Fig. 1) into a model that captures all the functions of interest for P1 (most severely injured patients), from reception through resuscitation to definitive care. This focussed WAI model (Fig. 3) will allow future “what if” analyses to test the system, with respect to P1 patients. Such “what if” scenarios include what if the number of patients presenting to the hospital exceeds the number of resuscitation bays.

### What did the FRAM model reveal about resilient MCI management?

The in-depth interviews supported by the FRAM model provided further insights into how the functions supported resilient operations during the management of the MCI. The function “To be surgical commander”, can be observed to provide the resilience potential to monitor and anticipate throughout the system during the incident. This key function was itself a spontaneous adaptation to practice by a single surgeon, which was not detailed in the major incident plan in advance of the incident. In practice the function was achieved by having a senior surgeon on the “shopfloor,” directly observing how care was being provided, as opposed to being sited in a command centre. A practical sequelae of FRAM modelling of the response is that this function has now been established in the major incident plan for a group of hospitals that includes the paediatric hospital studied. A second key function can be identified with many outputs, that of “To review as part of a multi-disciplinary team”. In addition to monitoring patients and ensuring holistic care to children and families in the ward setting, this function also enhanced resilience by capturing learning during the incident, in terms of extent of potential injuries, occult injuries to hunt for, understanding of potential human cross contamination due to shrapnel and other factors [4]. Noticeable by its absence in the model is a function to anticipate the number of incoming patients, which could have been achieved by establishing close contact with the scene of the incident. Review of all interview transcripts highlighted the lack of communication from the scene into the hospital.

A further key function is that of “To decide the patient destination”, this enhanced the response resilience potential of the system, in terms of damage control surgery, further damage control resuscitation or normal critical care / ward care. Further examination of how and where this function was achieved is warranted; particularly when one considers the one-way system of resuscitation-radiology-decision-making to Theatres or PICU, where it was imperative that patients did not return to the resuscitation bay after leaving for radiology.

The modelling has also highlighted the central and rate-limiting role of CT scanning has on the response of the system during the major incident. Due to the high likelihood of blast injuries, a high number of children were CT scanned. For the most seriously injured this was after approximately thirty minutes of resuscitation (including intubation, ventilation, and sedation) prior to CT in one of the three staffed resuscitation bays initially available. The model highlights the key function of “To perform CT scan”, particularly when the hospital has only one scanner available. If, unlike on the night of the event, more than three patients had arrived contemporaneously, each requiring resuscitation, then a second function of “To re-triage for CT during resuscitation” would be required to ensure the finite resource of the CT scanner was not targeted to the wrong patients during the one-way resuscitation flow described above. At the research site hospital this would now entail Trauma Team Leaders in the resuscitation bays having a structured communication huddle, possibly mid-stabilisations, to determine the appropriate order of patients to go to CT scan. This could mean that the first child stabilised for CT may not be the first to go, for example, if a patient with a time-critical head injury was “about” to be stable for CT scan, they would take priority.

Analysis of the expected mean process timings from FRAM model and mean actual process timings of the MCI (Table 2) also provided some other valuable insights for future MIP development. The model predicted that on average a patient would arrive in the CT scanner every 37 min. The actual average time was 38 min from arrival to commencing scanning, providing some construct validity to the modelling process. However, based on a damage control operative time of sixty minutes, analysis of the model highlights that actual times in theatre were more than twice this. It has been recognised that improvements in damage control timing is required, with a second anaesthetist in theatre now monitoring this in the current MIP. Comparison of the model and actual timings also shows a significant overshoot on predicted timings for post-operative PICU bed availability after the end of surgery, which is also being optimised.

### Limitations

A team experienced in creating and analysing FRAM models is required [16] and the structure and output of a FRAM model is dependent on the information provided to this team [17]. Reflexivity is a state of continual awareness and understanding on the part of research team members that their prior experiences and/or assumptions may influence all aspects of the study [18]. One researcher (RM) worked within the system modelled during the mass casualty event. Several steps were taken to foster a reflexive research study design including a continued reflexive dialogue between the international researchers with differing research backgrounds and understandings of the study phenomenon. While the model in this study was verified using the FRAM Model Interpreter and by comparing actual timings with model predictions, these methods may not capture all aspects of system performance, particularly under extreme stress conditions like MCIs. Also, the transferability of the FRAM model itself is limited since it is built on a local system. However, modern MCI management is built on similar principles in different settings and at that level the model might provide insights also in other health care settings.

## Discussion

The FRAM approach has identified how a paediatric trauma system functioned in response to the Manchester Arena Attack. The observed and reported resilient response of the paediatric hospital [4] is directly related to the system’s ability to monitor, anticipate, learn, and respond [7], both during and after the mass casualty incident. The functions identified in the model and how these functions are interconnected by couplings, provide key insights into the behaviour of the system.

This understanding of how variability within a system alone, or in combinations, may impact care provision is key to allow an after-action review of resilience. In this way the FRAM model is providing visual evidence akin to the descriptive evidence of a verbal, reflection on-action review experiential learning framework, which is well established in the debriefing literature [19]. Such a review of the major incident using the FRAM model in this way, allows further development of the above-described resilience potentials in advance of any future major incidents. Which in turn can facilitate re-design of a Major Incident Plan (MIP), as has happened at the study site.

Having an established, verified and internally validated a FRAM model of a MCI response, one can then advance further. It should then be possible to provide potential insights into the factors that promote resilience in healthcare systems exposed to extreme perturbations, that are key to policy makers and health care managers conducting risk and vulnerability analyses [20, 21]. Each FRAM function is in essence a mathematical equation describing the requirements to pass from its input stage to its output stage [10]. With each function being described by its own aspects (Timings, Controls, Preconditions required, and Resources used up) [9], variability can be introduced qualitatively into these aspects of each function in the model, for example “Too early”, “Too late” or “On time”, or “Precise”, “Imprecise” or “Not at all” [9]. The FRAM model directly allows the visualisation of such downstream consequences of upstream changes in the system. One can also introduce variability deliberately or observe the consequence of spontaneous adaptations to practice in the management of specific scenarios [15]. This Structured What-If FRAM approach has been utilised to observe the impact of approximate adjustments or adaptations to practice in trauma care on established key performance indicators [15]. As such one can introduce functions directly into this model and theoretically test and visualise the impact of the change to system.

As the FRAM model is in essence a cloud of inter-connected mathematical equations, it is now possible to explore variability quantitatively by inputting numerical “Metadata“ instead of the above qualitative data for the aspects of each function in the system. This numerical data can be inputted from clinical incidents or from developed fictional data specifically designed to stress test the system. An example of how this can be achieved is by cycling the FRAM model until finite resources; for example, available operating theatres, staff or PICU beds are used up. With this meta-data approach, it becomes possible to use the FRAM model to predict downstream impacts of upstream variability [22]. Moreover, one can start to stress the system and observe how it responds in different scenarios [22]. It is also now possible to explore the resilience of the system further utilising metadata with the resilience potentials used directly in the system in the form of functions “To respond”, “To monitor”, “To anticipate” and “To learn” as described by Nomoto [22], and then running consecutive iterations of the model to mathematically observe how the data variability may directly impact the system’s ability to maintain its resilience [22]. This approach will constitute the next stage of our research utilising the simplified WAI model (Fig. 3) to explore how the resilience of a paediatric major trauma system responds to differing conditions.

Where previously a “factory physics” approach of calculating how long a function takes and how many resources are used up, for example in a tabletop exercise, now the cycling FRAM model can potentially explore these scenarios. With a verified mathematical model, a series of Emergency Preparedness Resilience and Readiness (EPRR) scenarios could be explored, visualised and findings summated, stored and then easily disseminated electronically to a hospital wide audience to enhance learning. The mathematical programming of FRAM continues to evolve. Advances in FRAM now explores the mathematical modelling of queuing of patients arriving within a short time, modelling of overflows of patients into different directions within a model or the diversion to other functions based on probabilistic parameters. Where currently during a tabletop exercise with cards or figurines as patients, there is a shared imagination of the perturbation to the system of care during an MCI between those involved in the simulation; it is envisaged that FRAM may offer a computer-generated visual representation of perturbations and system responses as they occur in an MCI.

This represents an important step forward in EPRR analyses, as we postulate that this stepwise approach of FRAM may be applied to any healthcare system and any perturbations to such system.

## Conclusion

In conclusion, this application of FRAM has resulted in the creation of a model of how a paediatric Major Trauma Centre responded to a mass casualty incident. This model has facilitated an exploration of the intrinsic ability of a system to adjust its functioning during a major disturbance and provided key insights on how a resilient performance was sustained. The model serves an abstract construct, which with ongoing advances in FRAM; including infinite cycling, to use up resources and conditional situations producing different endpoints, now affords the opportunity for further research on the effects of a myriad of future potential perturbations to the system. Such modelling of complex adaptive healthcare systems is novel and may provide an important step forward in the preparedness and planning for future mass casualty incidents.

## Data Availability

The datasets used and/or analysed during the current study are available from the corresponding author on reasonable request.

## Abbreviations

FRAM: Functional resonance analytical methodology
MCI: Mass casualty incidents
MTC: Major trauma centre
PED: Paediatric emergency department
PICU: Paediatric intensive care unit
WAI: Work as Imagined
WAD: Work as done
HDU: High dependency unit
PCC: Paediatric critical care
CT: Computer tomography
EPRR: Emergency preparedness resilience and readiness
